# Potassium channel activity controls breast cancer metastasis by affecting β-catenin signaling

**DOI:** 10.1038/s41419-019-1429-0

**Published:** 2019-02-21

**Authors:** Eun-Kyoung Breuer, Daniela Fukushiro-Lopes, Annika Dalheim, Miranda Burnette, Jeremiah Zartman, Simon Kaja, Claire Wells, Loredana Campo, Kimberly J. Curtis, Ricardo Romero-Moreno, Laurie E. Littlepage, Glen L. Niebur, Kent Hoskins, Michael I. Nishimura, Saverio Gentile

**Affiliations:** 1 Department of Radiation Oncology, Loyola University Chicago, Stritch School of Medicine, Maywood, IL 60153 USA; 2 Department of Molecular Pharmacology & Therapeutics, Loyola University Chicago, Stritch School of Medicine, Maywood, IL 60153 USA; 3 Department of Surgery, Loyola University Chicago, Stritch School of Medicine, Maywood, IL 60153 USA; 4 Department of Chemical and Biomolecular Engineering, University of Notre Dame, Notre Dame, IN 46556 USA; 5 Department of Ophthalmology, Loyola University Chicago, Stritch School of Medicine, Maywood, IL USA; 6 Research Service, Edward Hines Jr. VA Hospital, Hines, IL USA; 7 Division of Cancer Studies, King’s College London, Rm. 2.34 A New Hunts House, Guy’s Campus, London, SE1 1 UL UK; 8 Harper Cancer Research Institute, University of Notre Dame, Notre Dame, IN 46556 USA; 9 Bioengineering Graduate Program, University of Notre Dame, Notre Dame, IN 46556 USA; 10 Division of Hematology Oncology, Department of Medicine, University of Illinois Chicago, Chicago, IL 60612 USA

## Abstract

Potassium ion channels are critical in the regulation of cell motility. The acquisition of cell motility is an essential parameter of cancer metastasis. However, the role of K^+^ channels in cancer metastasis has been poorly studied. High expression of the hG1 gene, which encodes for Kv11.1 channel associates with good prognosis in estrogen receptor-negative breast cancer (BC). We evaluated the efficacy of the Kv11.1 activator NS1643 in arresting metastasis in a triple negative breast cancer (TNBC) mouse model. NS1643 significantly reduces the metastatic spread of breast tumors in vivo by inhibiting cell motility, reprogramming epithelial–mesenchymal transition via attenuation of Wnt/β-catenin signaling and suppressing cancer cell stemness. Our findings provide important information regarding the clinical relevance of potassium ion channel expression in breast tumors and the mechanisms by which potassium channel activity can modulate tumor biology. Findings suggest that Kv11.1 activators may represent a novel therapeutic approach for the treatment of metastatic estrogen receptor-negative BC. Ion channels are critical factor for cell motility but little is known about their role in metastasis. Stimulation of the Kv11.1 channel suppress the metastatic phenotype in TNBC. This work could represent a paradigm-shifting approach to reducing mortality by targeting a pathway that is central to the development of metastases.

## Introduction

Breast cancer (BC) is a heterogeneous disease both biologically and clinically^1^. Tumor biology and clinical outcome are heavily influenced by the expression of proteins involved in estrogen-dependent signaling and the human epidermal growth factor receptor, type 2 (HER2) signaling pathway. Therapeutic strategies that target the estrogen receptor (ER) and HER2 signaling have improved survival for patients with ER-positive and HER2 over-expressing BC^2^, but tumors that do not express these proteins (so-called triple negative breast cancer, TNBC) often have a poor outcome. There is an urgent need for molecularly targeted therapies for the aggressive TNBC subtype.

All living cells are electrically polarized owing to a variety of ion channels and transport proteins in the cell membrane that control intracellular ion concentrations. Transmembrane ionic gradients determine membrane excitability, which regulates important cellular events including generation and transmission neuronal electrical signals and muscle contraction^3,4^. Recent studies show that the activities of several ion channels are associated with cellular migration and proliferation^5–10^. For example, potassium (K^+^) channels can control the phenotypic switch from an epithelial state to a mesenchymal phenotype (epithelial–mesenchymal transition; EMT)^11,12^, leading to loss of cell–cell contact and enhanced migratory and invasive capabilities^13,14^ in both physiologic states and pathologic conditions such as cancer. The human *KCNH2* gene encodes the voltage-dependent potassium (Kv) 11.1 channel, which is important for controlling membrane excitability^15^ and is abundantly expressed in various human cancers^16,17^. Studies show that expression of Kv11.1 during early stages of development is associated with the conversion of adherent epithelial cells into a mesenchymal phenotype^18^ and that uncontrolled gain or loss in Kv11.1 activity is often linked with tumor initiation and progression^19,20^.

Recently we reported that the *KCNH2* gene is overexpressed in several subtypes of BC and that treating ER-negative BC cell lines with molecules that activate the Kv11.1 ion channel (e.g., NS1643) induces cell cycle arrest^21–23^. In this study, we investigated the antimetastatic effect of the Kv11.1 channel activator NS1643 in vivo. For the first time, we demonstrate that pharmacologically activating the Kv11.1 potassium channel suppresses breast tumor metastasis in vivo and inhibits migration of ER-negative BC cells by reversing the EMT phenotype and cancer cell stemness. We show that the effect of NS1643 is mediated through the inhibition of β-catenin nuclear function, which in turn suppresses transcription of markers that are required for cellular migration. In silico analysis of patients with ER-negative BC supports the clinical significance of these findings. Our results identify a novel molecular mechanism by which activation of the Kv11.1 potassium channel suppresses BC growth and metastasis. These findings provide strong evidence to support the potential clinical application of Kv11.1 activators as targeted anticancer drugs for TNBC.

## Results

### NS1643-mediated stimulation of Kv11.1 activity inhibits breast tumor metastasis

**Fig. 1:**
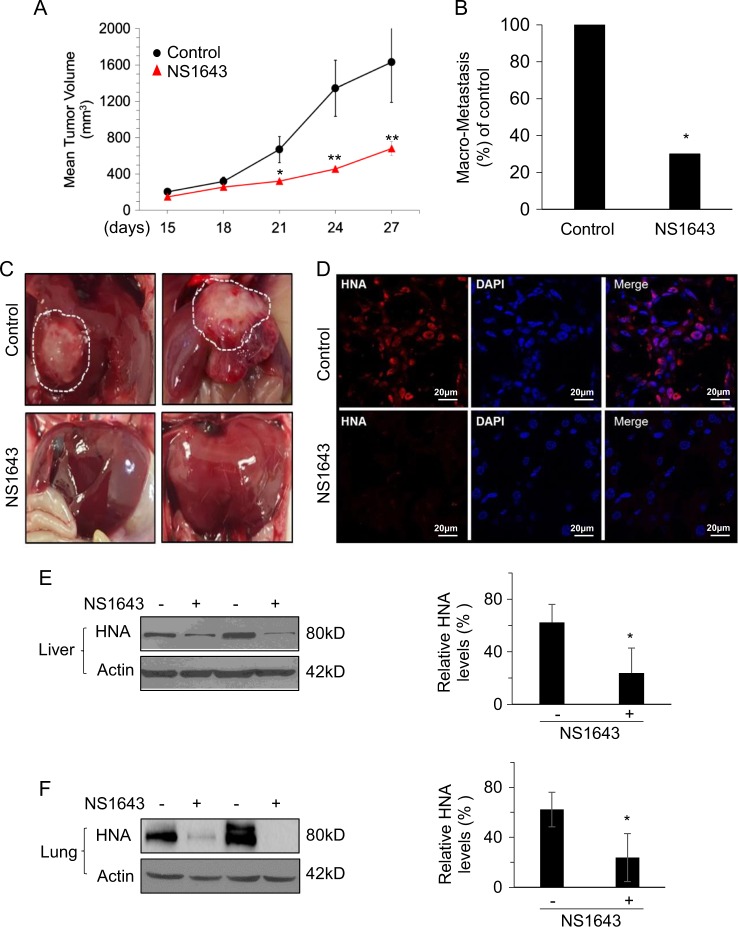
Kv11.1 stimulation inhibits primary tumor growth and metastasis in\ a xenograft model of breast cancer. MDA-MB-231 cells were injected subcutaneously into the dorsal flank of NSG mice. When tumors were palpable, the mice were injected intraperitoneally with vehicle alone or Kv11.1 activator NS1643 at 6 mg/kg every 2 days. **a** Mean tumor volume in mice treated with either vehicle control (*n* = 12) or Kv11.1 activator (NS1643) (*n* = 12). **b** Barchart showing the absolute frequencies of liver macrometastases observed in mice treated with either vehicle control or NS1643. **c** Representative images of livers demonstrating macrometastases in mice treated with either vehicle control or NS1643. **d** Representative confocal images of human nuclear antigen (HNA; red) and nuclei (DAPI; blue) expression in liver tissue sections from mice treated with either vehicle control or NS1643. **e** Representative western blot images of HNA expression in liver and (**f**) lung homogenates from mice treated with either vehicle control or NS1643. Quantification of the levels of HNA protein was done using NIH ImageJ software. Data is expressed as mean ± SEM; *p *<* 0.05; **p *<* 0.001)

In order to examine whether stimulation of Kv11.1 channel activity would inhibit BC growth and metastasis in vivo, we established human-derived TNBC xenograft tumors using MDA-MB-231 BC cells in NOD-scid IL2Rγ^null^ (NSG) mice^24^. MDA-MB-231 cells are known to express Kv11.1^21^ and to metastasize to distant organs including liver, lung, and bone following orthotopic injection into the mammary fat pad or subcutaneous injection into the flank of NSG mice^24^. As previously observed in nude mice, NS1643-treated NSG mice exhibited a persistent and significant reduction of tumor growth throughout the study compared with control mice. (Fig. 1a).

As expected, 25 days after subcutaneous injection of MDA-MB-231 cells into the dorsal rear flank we found evident macro-metastasis in the liver (Fig. 1b). When visible, metastatic liver tumors in the NS1643-treated group were significantly smaller than those in the control group (Fig. 1c). Control mice exhibited metastasis in both lobes of the liver, whereas liver metastases were confined to a single lobe of the liver in mice treated with NS1643. Furthermore, liver and lung tissue of mice treated with NS1643 displayed significantly decreased levels of human nuclear antigen (HNA), which labels human nuclei, compared with the control group (Fig. 1d, f).

To study the effect of NS1643 in a human metastatic tumor, we received a femoral head surgical specimen from a 59-year-old patient undergoing total hip arthroplasty owing to BC metastasis in the bone. We excised eight cylindrical trabecular bone explants which were cultured in custom in situ bioreactors for 6 days with or without 50 μm of NS1643 (Supplementary Figs. 1A and 1B). As expected, micro-computed tomography and histology showed disorganized bone, osteolysis, and the presence of tumor cells in all explants (Supplementary Fig. 1C–E). We have previously demonstrated that application of NS1643 strongly inhibits BC tumorigenesis by activating a senescence program. Interestingly, p16^INK4a^ protein expression, a marker of senescence, showed a trend toward higher expression in treated samples (Supplementary Figs. 1F and 1G). This ex vivo data generated with patient-derived metastatic breast tumor tissue confirms results obtained from BC cell lines, suggesting that activation of a senescence program induced by increased expression of p16^INK4a^ is one mechanism by which NS1643 inhibits tumor growth.

### Activation of Kv11.1 inhibits cell motility via suppression of EMT

**Fig. 2:**
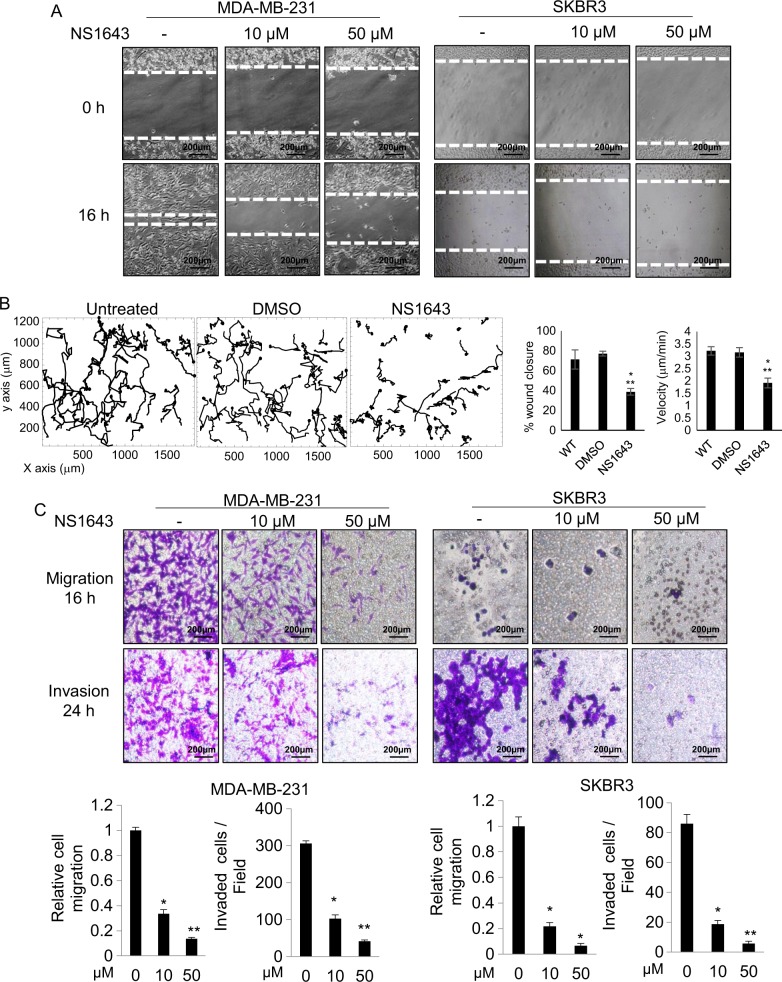
Kv11.1 stimulation inhibits breast cancer cell motility. **a** Representative images are showing the effect of Kv11.1 stimulation on breast cancer cell motility in wound healing scratch assays. Cells were treated with indicated concentrations of NS1643 (50 μm; *n* = 5; Dotted lines align with the center of the control border cell cluster). **b** Single-cell tracking of untreated control cells, vehicle control cells (DMSO) and NS1643-treated cells. Bar graphs show the effect of NS1643 on wound closure. Data are mean values ± S.E; *p < 0.01 or **p < 0.05 from WT and on cell speed. Data are mean values ± S.E; *p < 0.0001 or **p < 0.0001 from WT. **c** Representative images of the effect of Kv11.1 activator on breast cancer cell migration and invasion. Transwell migration and invasion assays were carried out. Student’s *t* test is used to compare two samples and analysis of variance with multiple testing corrections should be performed for comparing three or more groups of data. A *P* value < 0.05 is used to define statistical significance^82^

As NS1643 inhibited breast tumor growth and metastasis (Fig. 1) and because the metastatic potential is often associated with enhanced cell motility^25^, the inhibitory effect of NS1643 on cell migration and invasive capabilities was further examined with MDA-MB-231 (TNBC) and SKBR3 (HER2-positive) cell lines. Interestingly, NS1643 rapidly inhibited cell migration and invasion in a dose-dependent manner (Fig. 2a, b, c; Supplementary movie 1).

The Kv11.1 potassium channel is conserved in the fruit fly *Drosophila melanogaster*^26,27^. In the adult *Drosophila* ovary, a cluster of somatic cells known as border cells, detach from the epithelium and undergo invasive migration as part of normal development, and this event has been widely used as a model for studying cell migration and metastasis^28^. We took advantage of this model to test the effect of NS1643 on cell migration and found that NS1643 strongly inhibited migration of border cells (Supplementary Fig. 2; Supplementary movie 2).

**Fig. 3:**
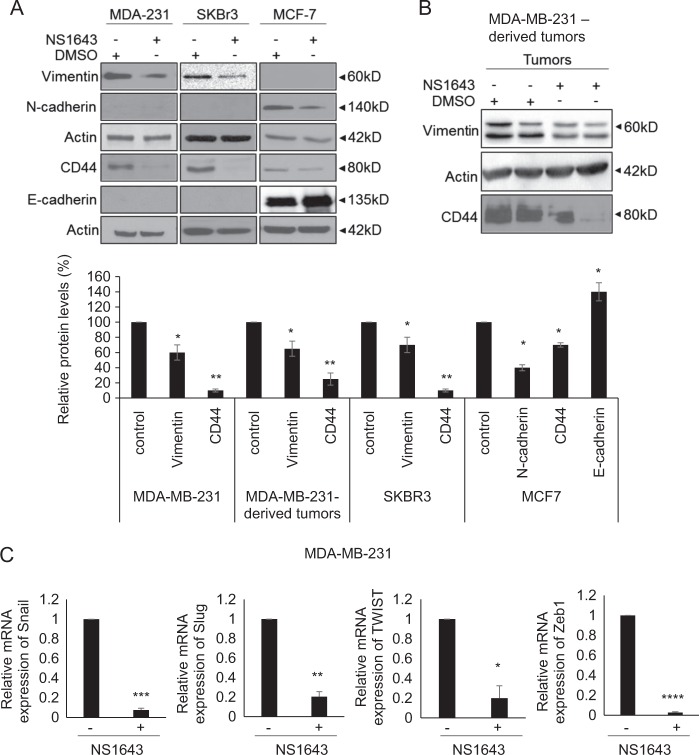
Kv11.1 stimulation inhibits reprograms EMT. **a** Representative western blot images of EMT markers (N-cadherin, Vimentin, CD44, and E-cadherin) in MDA-MB-231, SKBR3, and MCF7 breast cancer cells treated with either vehicle control or NS1643. Cells were treated with 50 μm of NS1643 for 24 h, harvested and subjected to western blot analysis. Actin was used as a loading control. Quantification of the levels of Vimentin, CD44, N-Cadherin, and E-cadherin was done using NIH ImageJ software. Data are expressed as mean ± SEM; *p *<* 0.005; **p *<* 0.001; *n* = *3*). **b** Representative western blot images of EMT markers, Vimentin and CD44, in MDA-MB-231-derived tumors from mice treated with either vehicle control or NS1643. **c** The mRNA levels of Snail, Slug, Twist, and Zeb were represented relative to β-actin transcripts. Cells were treated with 50 μm of NS1643 for 24 h, harvested, and subjected to qRT-PCR analysis. Data are expressed as mean ± SD of three independent experiments. *p *<* 0.05; **p *<* 0.01; ***p *<* 0.001; ****p *<* 0.00005

EMT is described as a loss of the stationary epithelial phenotype and upregulation of motile mesenchymal markers and has been shown to be significantly associated with increased cancer metastasis^29^. As stimulation of Kv11.1 channel activity suppresses migration and invasive capabilities of BC cells in vitro (Fig. 2a, b) and tumor metastasis in vivo (Fig. 1), we examined whether NS1643 has suppressive effects on the EMT process. We found that NS1643 led to a decrease in mesenchymal markers Vimentin, N-cadherin, and CD44, whereas it increased expression of the epithelial marker E-cadherin in BC cell lines (Fig. 3a). We also observed reduced expression of Vimentin in NS1643-treated mouse xenograft tumors derived from MDA-MB-231 (Fig. 3b). Finally, NS1643 significantly suppressed the expression of EMT-promoting transcription factors Snail, Slug, TWIST, and Zeb1 (Fig. 3c). These data suggest that activation of KV11.1 inhibits BC cell motility through suppressing EMT. Taken together, our data suggest that NS1643-mediated stimulation of Kv11.1 activity inhibits metastatic breast tumor growth, at least in part, by suppressing EMT and tumor cell motility.

### NS1643-mediated stimulation of Kv11.1 activity suppresses cancer cell stemness

**Fig. 4:**
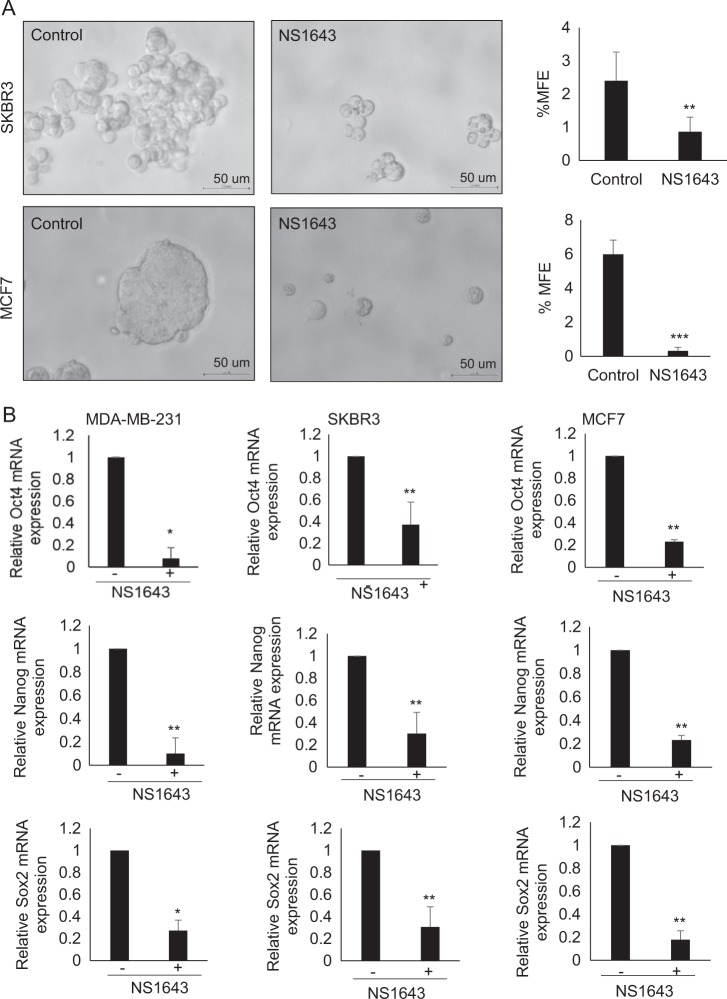
Kv11.1 stimulation suppresses the stemness of breast cancer cells. **a** Representative images are showing the effect of Kv11.1 stimulation on mammosphere forming efficiency (MFE) in SKBR3 and MCF7. Cells were seeded into Ultra-low attachment 6-well plate under DMSO or 50 μm of NS1643 for 7 days. **b** The mRNA levels of Oct4, Nanog, and Sox were represented relative to β-actin transcripts. Cells were treated with 50 μm of NS1643 for 24 h, harvested, and subjected to qRT-PCR analysis. Data are expressed as mean ± SD of three independent experiments. *p *<* 0.01; **p *<* 0.05; ***p *<* 0.0005

Mounting evidence indicates that EMT is closely associated with cancer cell stemness characteristics^30,31^. As CD44 is a stemness marker and is known to be associated with EMT^32^, and because we found that the expression of CD44 was reduced by NS1643 treatment (Fig. 3a & b), we speculated that NS1643 suppresses EMT, which in turn attenuates cancer stemness, thereby inhibiting breast tumor growth and metastasis. To further investigate the effect of NS1643 on stem cell-like characteristics, we carried out mammosphere formation assays. NS1643 treatment significantly reduced the mammosphere formation capability of SKBR3 and MCF7 cells (Fig. 4a). We then examined the expression of stem cell-specific transcription factors Oct4, Nanog and Sox2^33^, and found that the expression of all of these markers was significantly reduced by NS1643 treatment (Fig. 4b). These data indicate that NS1643-mediated stimulation of Kv11.1 activity suppresses the stemness of BC cells.

### NS1643 inhibits β-catenin transcriptional activity and increases cell–cell contact

**Fig. 5:**
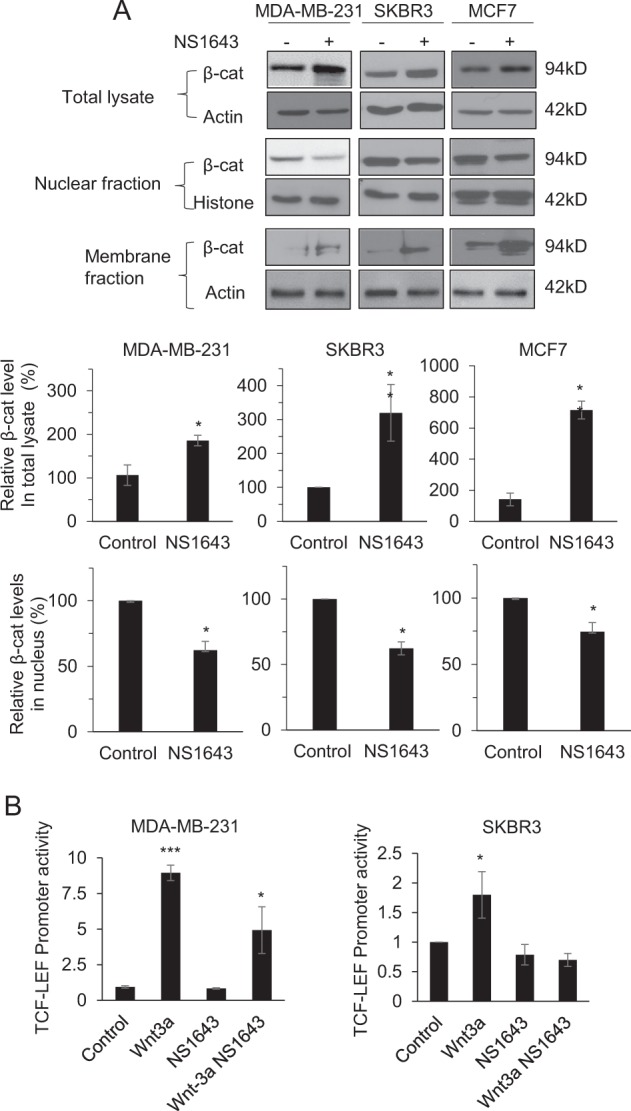
Kv11.1 stimulation regulates the cellular localization of β-catenin. **a** Representative western blot images are demonstrating subcellular localization of β-catenin. Cells were treated with 50 μm of NS1643 for 24 h, harvested, and subjected to cellular fractionation (nuclear and membrane fraction). Quantification of the levels of β-catenin protein was done using NIH ImageJ software. *p *<* 0.05; **p *<* 0.005; ***p *<* 0.001 **b** β-catenin TCF/LEF promoter activities in control group, NS1643-treated group, Wnt3a-treated group (positive control) and NS1643 + Wnt3a-treated group were measured using TopFlash and FopFlash vectors

Accumulating evidence indicates that the Wnt/β-catenin signaling pathway has an essential role in the regulation of EMT process and cell motility in cancer^34^. β-catenin has the dual function of coordinating cell–cell adhesion when it colocalizes with E-cadherin at the surface membrane or promoting cell migration when it localizes to the nucleus where it functions as a transcription factor in the Wnt signaling pathway^35^. As NS1643 suppresses EMT and cell motility, we examined the expression, subcellular localization, and transcriptional activity of β-catenin in BC cell lines following treatment with NS1643. Interestingly, NS1643 resulted in the elevation of total β-catenin expression (Fig. 5a). Subcellular fractionation analysis of β-catenin revealed that NS1643 causes an apparent reduction in nuclear β-catenin levels and thus cytosolic accumulation (Fig. 5b. Using TOP-Flash/FOPFlash reporter assays, we found that NS1643-mediated nuclear export of β-catenin slightly reduced the basal level of transcriptional activation of β-catenin, but it significantly decreased β-catenin transcription following Wnt3a stimulation (Fig. 5b).

**Fig. 6:**
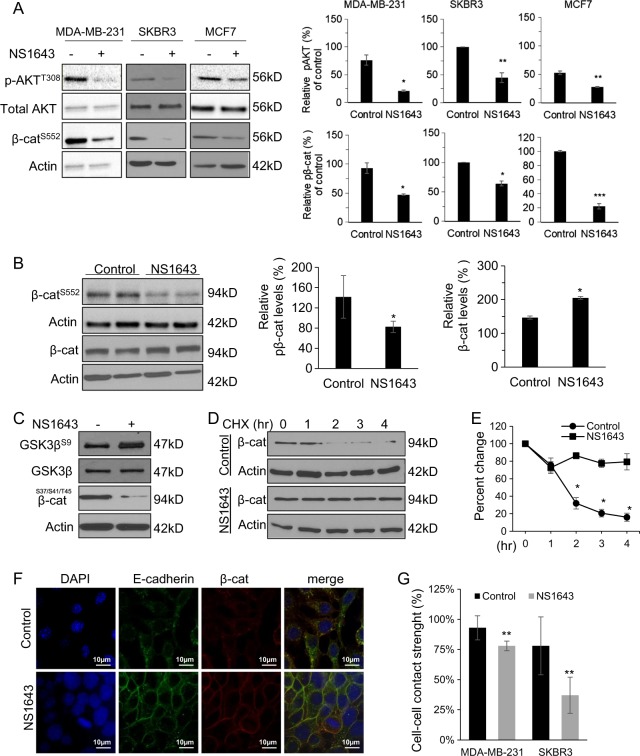
Kv11.1 controls β-catenin nuclear translocation signaling. **a** Representative western blot images of phospho-AKT^T308^, phospho-β-catenin^S552^, and total AKT levels in cells or **b** tumors extracted from mice treated with control or NS1643. Cells were treated with 50 μm of NS1643 for 24 h, harvested, and subjected to western blot analysis. Actin was used as a loading control. Quantification of the levels of phospho-AKT and phospho-β-catenin were done using NIH ImageJ software. **c** Representative western blot images of phospho-GSK3⍰Ser9 and total GSK3⍰ levels in MDA-MB-231. **d** Representative western blot images of β-catenin levels in cells treated with cycloheximide alone or NS1643 for the indicated time and **e** quantification of the effects (*n* = 3). Quantification of the levels of β-catenin and phospho-β-catenin were done using NIH ImageJ software. **f** Confocal microscopy images are representing the accumulation of β-catenin at the surface membrane in MDA-MD-231 cells treated with control vehicle or NS1643 for 24 h. **g** Graph showing the effect of NS1643 on cell–cell contact measured by Dispase assay (*n* = 6; mean ± SD; *P < 0.05)

AKT-mediated phosphorylation of β-catenin on Ser 552 promotes nuclear translocation and transcriptional activation of β-catenin, thereby enhancing metastasis^36^. We found that NS1643 inhibited activation of AKT, indicated by decreased phosphorylation of both AKT on T308 and β-catenin on Ser 552 in cell lines (Fig. 6a) and MDA-MB-231-derived tumor xenografts (Fig. 6b).

GSK3β-dependent phosphorylation of β-catenin promotes β-catenin degradation. We discovered that NS1643 inhibited GSK3β activity as indicated by the increased phosphorylation of Ser9 on GSK3β and by the decreased phosphorylation of the GSK3β target sites on β-catenin). Consequently, degradation of β-catenin was significantly inhibited by NS1643 (Fig. 6c–e). Importantly, we found that NS1643 promoted localization of β-catenin to the surface membrane and increased cell–cell contact (Fig. 6a, f, g).

### Expression of *KCNH2* is associated with improved survival in patients with ER-negative BC

**Fig. 7:**
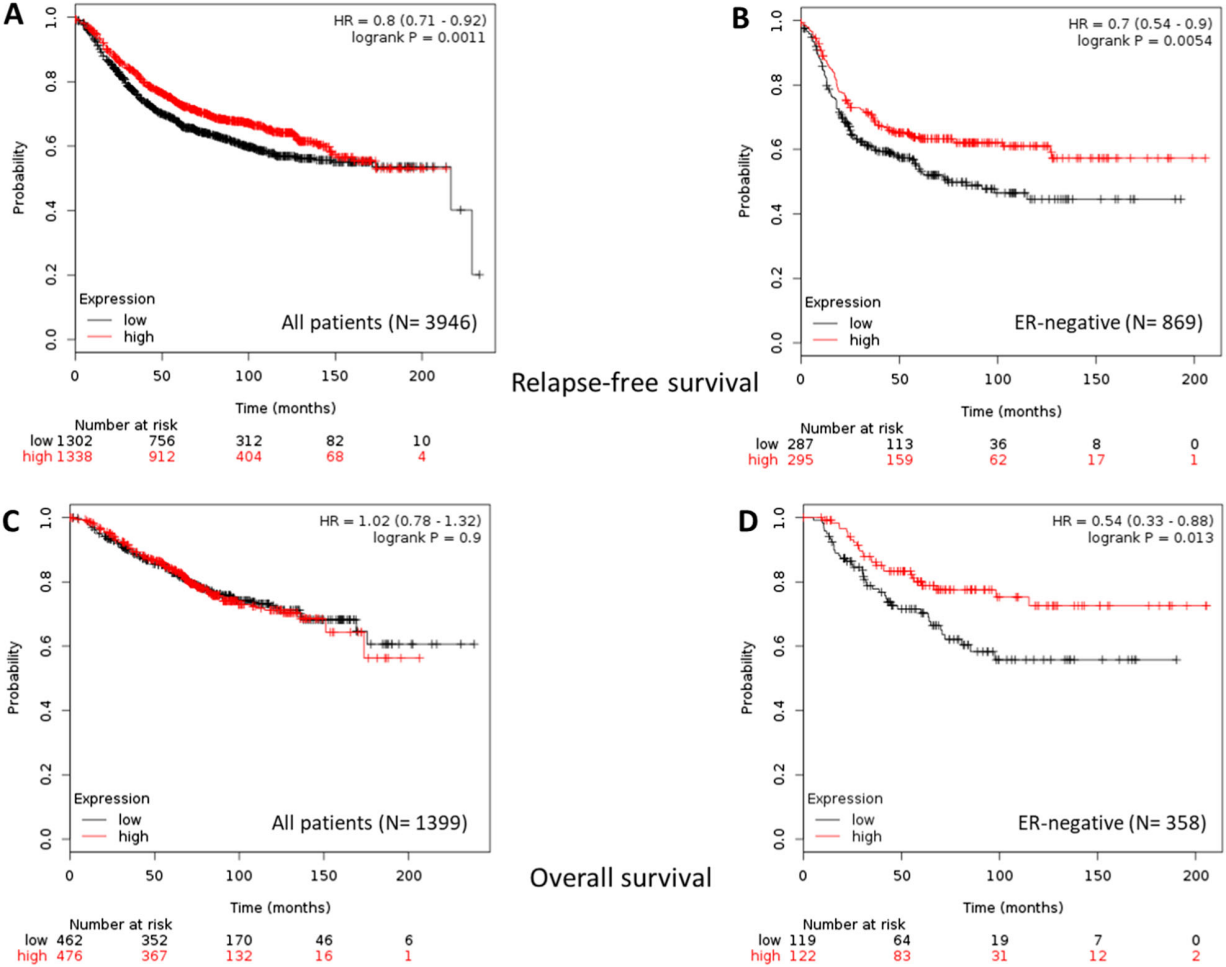
KCNH2 expression and survival in patients with breast cancer. Kaplan–Meier plots of relapse-free survival **a**, **b** and overall survival **c**, **d**, according to the level of *KCNH2* gene expression (top vs. bottom tertiles). Results are presented for all breast cancer patients **a**, **c** and the estrogen receptor-negative subgroup **b**, **d**. Hazard ratios (HR) compare the hazard of relapse or death in the high expression vs. low expression groups

To explore the clinical relevance of our findings, we conducted in silico analyses^37^ investigating the association of *KCHN2* gene expression with survival in BC patients. Results of a meta-analysis of *KCNH2* expression showed that high expression was significantly associated with improved relapse-free survival (HR = 0.8; 95% CI, 0.71–0.92, Fig. 7a). Subgroup analysis revealed that the association was seen only in ER-negative patients (Fig. 7b), with a HR = 0.7; 95% CI, 0.59–0.9 (compared with HR = 0.89; 95% CI, 0.76–1.04, *p* = 0.13 in the ER-positive subgroup). Similarly, only ER-negative patients showed an association of *KCNH2* expression with overall survival (Fig. 7d) with HR = 0.54 (95% CI, 0.33–0.88, *p* = 0.013). These findings support in vivo xenograft experiments demonstrating that activation of the Kv11.1 channel primarily affects the development of metastases in tumors derived from ER-negative BC cell lines.

**Fig. 8:**
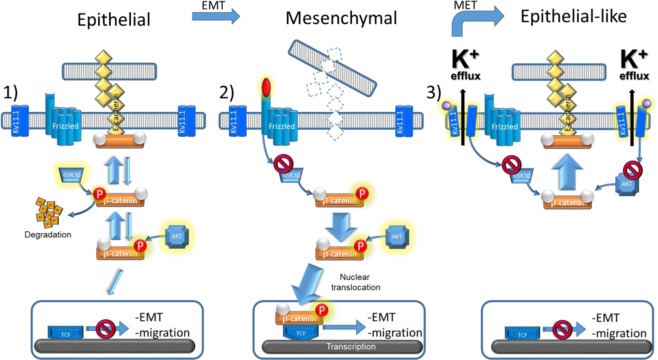
Schematic representation of the antimetastatic signaling promoted by activation of Kv11.1. 1) In normal epithelial cells, β-catenin is in complex with adhesive proteins (e.g., E-cadherin) and promotes cell–cell contact. GSK3⍰-dependent phosphorylation controls β-catenin degradation while AKT-dependent phosphorylation controls β-catenin nuclear translocation. 2) In cancer cells, the Wnt-dependent signaling arrests β-catenin degradation by inhibiting GSK3β activity. Consequently, upon AKT phosphorylation β-catenin translocates to the nucleus and transcribes for genes controlling epithelial to mesenchymal transition (EMT) and migration. 3) Activation of the Kv11.1 promotes epithelial phenotype in mesenchymal cancer cells (MET) by inhibiting GSK3β-dependent β-catenin degradation and AKT-dependent nuclear translocation of β-catenin, producing β-catenin accumulation at the surface membrane and therefore, improving cell–cell contact

Together, our data suggest that stimulation of Kv11.1 channel activity simultaneously inhibits β-catenin nuclear translocation and thus downregulates its transcriptional activity, and upregulates its surface membrane localization thereby increasing cell–cell contact (Fig. 8).

## Discussion

Distant metastases are the primary cause of cancer-related death. Despite therapeutic advances leading to improved survival for patients with early BC, development of novel and effective therapeutic strategies to overcome treatment resistance in advanced BC remains a critical unmet need^38^. Our novel findings, which convincingly demonstrate the potential of Kv11.1 channel activators to suppress the metastatic phenotype in ER-negative BC, take on added significance given the absence of FDA-approved targeted therapies for advanced TNBC.

During normal biological processes such as embryonic development and wound healing, cells lose polarity and adhesion to surrounding cells and transition into a mesenchymal phenotype with a gain in migratory properties^39^. Mounting evidence indicates that ion channels are associated with EMT^11^. For example, Kv11.1 has a critical role during the development of the neural tube, which requires cells to undergo EMT and migrate to defined destinations^18^. This process is replicated in neoplastic cells capable of metastasizing, yet the role of specific ion channels in generating a metastatic phenotype is still poorly understood.

Our previous work and others demonstrated that Kv11.1 channel is expressed in a variety of BC cells but not in normal breast cells^21,40^. Interestingly, use of the Kv11.1 activator NS1643 strongly inhibited tumor growth by activating a cellular senescence phenotype in BC cell lines. In contrast, NS1643 did not produce any effect in normal breast cells. In this study, we confirmed that NS1643 also produces an inhibitory effect on tumor growth in a human-derived cell line xenograft model of TNBC. Furthermore, NS1643 produced an overall increase in the expression of the senescence marker p16^INK4A^^41^ in a human BC bone metastasis ex vivo model. Unfortunately, owing to the limited availability of human samples, this investigation was restricted to a single patient. However, these data suggest that NS1643 suppresses the metastatic phenotype of human BC.

We and others reported that use of Kv11.1 blockers or gene suppression produces cytotoxicity in several cancer cells^42–45^ but the use of Kv11.1 blockers as therapeutic agents is discouraged due to their negative effects on the heart^19^ (e.g., ventricular fibrillation). In contrast, Kv11.1 activators are well tolerated in mammals^46,47^. Potential Kv11.1 activator-dependent side effects might include tachycardia^46^. However, this clinical condition is easily correctable by the routine measure of intervention (e.g., beta-blocker) during treatment of patients with cancer and, in comparison to the side effect of current antimetastatic agents which are often fatal^48–51^ (e.g., doxorubicin-dependent congestive heart failure), presents a minimal impact on patients health. Therefore, the potential benefits of Kv11.1 activators as anticancer drugs outweigh their side effects. Also, we have established that chemically distinct Kv11.1 activators present similar effects on cancer cells. Nevertheless, they do not elicit any measurable response if applied to non-carcinogenic BC^21,23,52^ or other epithelial cells that do not express Kv11.1. Furthermore, the inhibitory effect of NS1643 on BC cell proliferation is at least partially reversed by the application of channel blockers such as E4031^21^. Nevertheless, because drugs these molecules have shown some degree of promiscuity as they can target different potassium channels^53,54^ we cannot completely exclude that the effects of these molecules is solely mediated by the intendent target. Nevertheless, in view of the fact that opening of potassium channels always drives an outward flow of K^+^ ions, we can conclude that targeting K^+^ channels with activator molecules could be considered an anti-proliferative strategy.

Although senescent cells are hypo-proliferative, they are metabolically active and potentially able to perform the function of the cell from which they derive^55^. Our in vitro cell migration assays demonstrated that NS1643 significantly inhibits the motility of BC cells as early as 12 h before any detectable changes in cell proliferation^21^. Our results are first to suggest that Kv11.1 stimulation inhibits both cancer cell proliferation and motility, thereby suppressing primary tumor growth and dissemination of distant metastases. In order to determine the mechanism by which Kv11.1 inhibits cancer cell motility, we pursued the regulation of EMT. The expression of EMT markers varied depending on the cell line tested, which is not surprising given the heterogeneity of BC. Following Kv11.1 activation, markers promoting mesenchymal phenotype including Vimentin, CD44, and N-cadherin were significantly reduced in ER-negative BC cells relative to the basal expression of these markers. Downregulation of Vimentin and CD44 in response to NS1643 was also observed in tumor xenografts derived from ER-negative cell lines. This event, in combination with the low incidence of metastasis to the livers and lung in NS1643-treated mice, supports the previous reports that expression of CD44 or Vimentin in breast tumors facilitates the development of pulmonary and hepatic metastases^56,57^.

Evidence shows that cancer cells undergoing EMT gain stem cell-like characteristics^30,31^. Pluripotency-associated transcription factors, Oct4, Sox2, and Nanog, are expressed in both embryonic stem cells and cancer stem cells^58,59^. The expression of Sox2 has been shown to be associated with mammosphere formation^60^ and breast tumor metastasis^61^. Studies show that simultaneous expression of Nanog and Wnt1 promotes cell motility, thereby enhancing breast tumorigenesis and metastasis in the mouse mammary gland^62^, and that high levels of Nanog predict poor survival in BC patients^63^. The expression of Oct4 is significantly associated with lymph node metastasis in BC patients^64^. In this study, we found that NS1643 suppressed cancer stem cell characteristics, providing additional support for the hypothesis that NS1643 inhibits primary breast tumor growth and metastasis by suppressing EMT and cancer cell stemness.

To determine the mechanism by which Kv11.1 activation regulates EMT, we investigated the effect of N1643 on the regulation of the Wnt/β-catenin pathway, which controls cell proliferation, migration, and differentiation involved in mammary development and oncogenesis^65^. Others have shown that hyperactive Wnt/β-catenin signaling is significantly associated with the progression of advanced metastatic TNBC and that enhanced nuclear accumulation of β-catenin is associated with poor prognosis^66^. Vimentin, CD44, and N-cadherin are downstream targets of the Wnt/β-catenin signaling pathway and are known to drive breast tumorigenesis^67^. Consistent with our result showing a marked decrease in EMT markers, NS1643 significantly reduced the levels of nuclear β-catenin and the nuclear activity of β-catenin.

Interestingly, in contrast to the significant decrease in nuclear β-catenin levels, NS1643 increased total levels of β-catenin. NS1643 stabilized β-catenin protein by inhibiting GSK3β phosphorylation. Consequently, β-catenin associates with E-cadherin, thereby promoting E-cadherin-mediated intercellular adhesion^68^ and converting cells to an epithelial phenotype. Despite lack of E-cadherin in MDA-MB-231 and SKBR3 cells, Kv11.1 stimulation also enhanced β-catenin-mediated intracellular adhesion, suggesting that β-catenin may bind to other cadherins such as cadherin-11, which is expressed on MDA-MB-231 cells^69^.

The studies reported here strongly suggest that, in addition to its classic role in controlling action potentials and membrane excitability in neurons and myocytes, the Kv11.1 potassium channel has a major role in cancer homeostasis, and that stimulation of this channel suppresses primary tumor growth and metastasis at least in part through activating cellular senescence program and inhibiting cell motility, EMT process, and cancer cell stemness. The effects appear especially prominent in ER-negative BC. Based on our findings, we propose that small molecule activators of the Kv11.1 channel represent a novel therapeutic strategy to inhibit tumor growth and metastasis in TNBC. Although the toxicity of these agents needs to be carefully studied, our early animal work suggests that these agents are well tolerated in doses that demonstrate the antitumor effect. Further work with preclinical models to more precisely define the activity, mechanisms of action and tolerance to treatment with this novel class of drugs is warranted.

## Materials and methods

### Tumor xenograft models

Two million MDA-MB-231 BC cells were subcutaneously injected (rear flank) in NSG mice (*n* = 12). Mice were randomly divided into two groups (*n* = 6/group) and treated with vehicle (DMSO) or NS1643 via intraperitoneally (i.p.) (NS1643 6 mg/Kg) twice per week. Tumor size was measured every 5 days and mice were killed 6 weeks after the initial NS1643 treatment. This study was reviewed and approved by Loyola University Chicago Institutional Animal Care and Use Committee guidelines (Animal Assurance No. D16-00074).

### Human metastatic bone culture

A femoral head from a 59-year-old patient undergoing total hip arthroplasty owing to BC metastasis in the bone was obtained from Loyola Medical Center. Eight cylindrical trabecular bone explants, 8 mm diameter by 10 mm long, were prepared from a human femoral head with existing metastatic BC. The explants were cultured in a custom bioreactor^70–73^ with 89% Dulbecco’s modified essential medium, 10% fetal bovine serum, and 1% antibiotic/antimycotic circulated at 0.9 ml/min with a peristaltic pump. Media was supplemented with 50 µm NS1643 in four explants. Following culture, the explants were fixed in neutral buffered formalin, imaged by µ-CT at 20 µm resolution (Scanco µ-CT 80, Brütissellen Switzerland). The fixed explants were demineralized for 4 weeks in neutral buffered EDTA, processed, and sectioned at 5 µm. Immunohistochemistry was performed as previously described^72,73^ with pan-cytokeratin (1:750) and p16^INK4a^ (1:100) antibodies. The fraction of the area exhibiting p16^INK4a^ staining was quantified in 20 regions (949 × 467 µm) of marrow in each explant using ImageJ (NIH). The Medical Ethics Committee of Loyola Medical University approved this study (IRB 15-04-2500). All the samples were anonymous.

### Cell culture, antibodies, and reagents

MDA-MB-231, SKBR3, and MCF7 human BC cell lines were obtained from the American Type Culture Collection (ATCC) (Manassas, VA) and maintained 231 cells (ATCC) was maintained in Dulbecco’s modified Eagle’s medium (DMEM) (4.5 g/L glucose) supplemented with 10% fetal bovine serum, Penicillin (100 µg/ml)/streptomycin (100 µg/mL) antibiotics. Cells were incubated under humidified conditions with 5% CO_2_ at 37 °C.

Antibodies against phospho-β-catenin^S552^, phospho-β-catenin^S33/S37/T41^, β-catenin, E-cadherin, N-cadherin, Vimentin, CD44, phospho-GSK3β^S9^, GSK3β, phospho-AKT^T308^, AKT, pan-cytokeratin and Actin were purchased from Cell Signaling Technologies, Inc (Boston, MA). An anti-HNA antibody was purchased from Abcam (Cambridge, UK). p16^INK4a^ was purchased from Abcam.

Cycloheximide (CHX) was purchased from Sigma-Aldrich (St. Louis, MO) and NS1643 was purchased from Alomone Labs (Jerusalem, Israel).

### Western blot analysis, immunoprecipitation, and immunofluorescence staining

Cells were harvested and lysed with cold radioimmunoprecipitation assay (RIPA) buffer (50 mm Tris HCl (pH 8.0), 150 mm NaCl, 1% NP-40, 0.5% sodium deoxycholate, 0.1% sodium dodecyl sulphate (SDS), 1 mm phenylmethylsulfonyl fluoride (PMSF), 1 mm sodium fluoride (NaF), 1 mm sodium orthovanadate (Na_3_VO_4_), and protease inhibitor cocktail). An equal amount of protein samples were subjected to SDS-polyacrylamide gel electrophoresis (PAGE) and transferred onto a nitrocellulose membrane. Membranes were blocked with 5% nonfat milk in Tris-Buffered Saline (TBS) containing 0.1% Tween 20 (TBST), incubated with primary and secondary antibodies, and detected using Super Signal West Pico Chemiluminescent Substrate (Thermo Scientific, Pittsburgh, PA). Immunoprecipitation was performed by incubating protein lysates with primary antibodies at 4 °C overnight, followed by incubation with protein A/G-conjugated agarose beads (Santa Cruz Biotechnology, Santa Cruz, CA). Beads were washed with cold RIPA buffer, resuspended in 2 × SDS sample buffer, boiled for 5 min, and subjected to SDS-PAGE, followed by western blot analysis. Cells were grown on poly d-Lysine coated coverslips and treated with or without 50 µm NS1643. Following treatment, cells were washed with 1 × phosphate-buffered saline (PBS), fixed with acetone:methanol fixation, blocked with 5% bovine serum albumin (BSA) for 1 h at room temperature, and incubated with primary antibodies, followed by incubation with Alexa Fluor 594- or Alexa Fluor 488-conjugated secondary antibody (Life Technologies, Carlsbad, CA). Next, slides were incubated with 50 ng/mL DAPI to stain the nuclei. The coverslips were mounted on VECTASHIELD reagent (Vector Laboratories, Burlingame, CA) and fluorescent images were taken using confocal microscopy (Carl Zeiss Meditec, Inc., Thornwood, NY). Cells incubated with 1% BSA alone were served as negative controls.

### Nuclear/membrane fractionation

Cells were washed, lysed with a hypotonic buffer (10 mmol/L 4-(2-hydroxyethyl)-1-piperazineethanesulfonic acid (HEPES) buffer (pH 7.9), 1.5 mmol/L MgCl_2_, 10 mmol/L KCl, 10% glycerol, 0.5 mmol/L PMSF, and 1 mmol/L dithiothreitol (DTT)), and incubated on ice for 15 min. Five μl of 10% NP-40 was added to each lysate. After centrifugation at 13,200 rpm for 10 min, the supernatant was collected as the membrane/cytosolic fraction. The nuclear pellet were lysed with buffer solution containing 20 mmol/L HEPES buffer (pH 7.9), 1.5 mmol/L MgCl_2_, 400 mmol/L NaCl, 0.2 mmol/L EDTA, 20% glycerol, 0.5 mmol/L PMSF, and 1 mmol/L DTT, and collected by centrifugation at 13,200 rpm for 10 min at 4 °C.

### Luciferase assay

Cells were co-transfected with plasmid encoding firefly luciferase driven by TCF-LEF promoter and a plasmid encoding a constitutively expressed Renilla luciferase gene with Lipofectamine 2000 transfection reagent. Twenty four hours after transfection, cells were treated with vehicle alone or NS1643 for 16 h. Cell lysates were analyzed for luciferase activity using a dual-luciferase assay system according to the manufacturer’s instructions (Promega, Madison, WI).

### Wound closure

MDA-MB-231 cells were plated onto 6-well plates. Cells were incubated for 24 h to allow a monolayer to form. Cells were scratched with a small pipette, 25 mm Hepes was added, and cells were treated immediately with 50 μm NS1643 or DMSO. The sealed plate was then placed on the automated heated stage of an Olympus IX71 microscope set at 37 °C and imaged with a 5× UPlanFLN objective lens. Images were collected using a Retiga SRV CCD camera, taking a frame every 5 min for 16 h from each of the wells using Image-Pro Plus software. Subsequently, all the acquired time-lapse sequences were displayed as a movie. Movies were tracked using ImageJ tracking plugin and analyzed using in-house Mathematica software as previously described^74^. Only edge cells were tracked for entire 16-hour movie—data are two independent experiments 30 cells tracked per condition.

### Scratch wound healing and transwell migration assays

Cells were cultured as confluent monolayers, and the scratch was generated using a sterile 200 μl pipette tip. Phase-contrast light micrographs were taken immediately after cell removal and at 16 and 24 h for analysis. Migration assays were performed using uncoated cell culture inserts (8  μm pore size, Greiner Bio-One, Monroe, NC). Cells were resuspended in serum-free culture media at a density of 10^6^ cells/ml in the absence or presence of NS1643 and added to the upper chamber while the bottom chamber was filled with serum-contained complete culture media. After 16 h of incubation, non-migratory cells on the top surface of the filter were scraped off with a cotton swab. Migrated cells on the bottom surface were fixed, stained with 0.05% crystal violet, and counted.

### Drosophila model for migration

Egg chambers from flies expressing E-cadherin: GFP were prepared and dissected as described previously^75^. Chambers were cultured using a slightly modified setup from Prasad, with 33 µg/mL human insulin, and using a Millicell® cell culture insert setup^76^ in lieu of the lumox culture dish. Time-lapse movies were acquired by confocal microscopy with eleven 4 µm z-sections taken at 15-mi intervals. The analysis was done in FIJI using egg chambers exhibiting normal morphology with a minimum duration of 1.5 h. Net cluster migration was based on starting and final x-position of the cluster center. For drug treatment, culture media above the culture dish was supplemented with either 50 µM NS1643 or vehicle (DMSO). Imaging was set up immediately after dissection with an average lead time of ~ 20 min.

### Invasion assay

Invasion assays were carried out using QCM ECMatrix Cell Invasion Assay kit (24-well, 8 μm, colorimetric) (EMD Millipore, Billerica, MA) according to the manufacturer’s instructions. Briefly, cells were suspended in serum-free media and added to the inserts. The lower chamber was filled with serum-contained complete media. After 24 h of incubation, the invaded cells were fixed, stained, and counted.

### Mammosphere formation assay

After trypsinization, 10^5^ cells in 2 ml of mammosphere culture medium with or without NS1643 were plated into ultra-low attachment plates and incubated at 37 °C for 7 days. The mammosphere culture medium is composed of 1.7 % methylcellulose, phenol red-free DMEM/F12 media with 1 × B27 supplement minus vitamin A and 20 ng/ml of EGF. After 7 days, pictures were taken with a phase-contrast light microscope at × 20magnification. The mammospheres were extracted by slowly breaking down the mammosphere media with increasing amount of PBS. The solution was spun down twice at 1000 rpm for 5 min. After removing the supernatant, the pellet was suspended in 1 ml of PBS. For mammosphere quantification, 50 μl of solution was aliquoted into one 96 well plate and pictures were taken at 4x magnification. The pictures were transferred into MS PowerPoint 2010 at a size of 7 × 9 .33 inches, and the letter O at a size of 29 Calibri bold was used to individualize mammospheres 50 microns or larger. The percentage of mammosphere forming efficiency was calculated with the formula: (number of cells counted × dilution factor)/number of cells plated × 100.

### Quantitative real-time-polymerase chain reaction (qRT-PCR)

Total RNAs were extracted from cells using RNeasy^®^ mini kit (Qiagen, Hilden, Germany). The reverse transcription was carried out with a RevertAid ^TM^ First Strand cDNA Synthesis Kit (Fermentas Life Sciences Europe, Bremen, Germany) using Oligo(dT) primers. For qRT-PCR analyses, an iCycler iQ5 real-time PCR detection system (Bio-Rad) and an iQ^TM^ SYBR^R^ Green Supermix (Bio-Rad) were used as follows: 95 °C for 5 min and 30 s, and 40 cycles (15 s at 95 °C, 1 min at 60 °C). The data were analyzed with a normalized gene expression method (ΔΔ Ct)^77^ using the iQ5 Optical System Software (Bio-Rad), and the β-actin was used as a reference for normalization. All the measurements were performed in triplicate. The sequences of the primer pairs were as follows:

*Snail* 5′-ttctctaggccctggctgctacaa-3′ and 5′-tctctgacatctgagtgggtctgga-3′;^78^

*Slug* 5′-ctggtcaagaagcatttcaacgcc-3′ and 5′-aaagaggagagag gccattgggta-3′;^78^

*TWIST* 5′-ggagtccgcagtcttacgag-3′ and 5′-tctggaggacctggtagagg-3′;^79^

Zeb1 5’-ctccagaatgtaatcgcatgtg-3’ and 3’-tgagaaaaccttatagaacagtgtg-5’;

*β-actin* 5′-ctacgtcgccctggacttcgagc-3′ and 5′-gatggagccgccgatccacacgg-3′^80^.

Primers for *Oct4, Sox2*, and *Nanog* were purchased from Santa Cruz Biotechnology.

### Dispase-based dissociation assay

Cell–cell adhesive strength was measured by a modified dispase assay^81^ as it follows: cells treated with DMSO (control) or NS1643 (50 μm) were washed in PBS and incubated with 0.6 ml of dispase (Roche) for 60 min. Then, the detached monolayer was washed and resuspended in PBS. Mechanical stress was applied by 20 pipetting with an automatic pipette (Eppendorf), and single cells were counted in a 10 μL aliquot. One well of each triplicate was treated with trypsin instead of dispase, to determine the total cell number, which was divided by the mechanically released single cells. Statistical analysis was performed on three separate experiments, and statistical significance (*t* test) was defined as *P* < 0.05 for MDA-MB-231 or *P* < 0.005 for SKBr3.

### Effect of *KCHN2* gene expression levels on patient survival

We performed a meta-analysis to explore the association of *KCHN2* gene expression with survival in BC patients using KM plotter (KMplotter.org). This web-based application uses a manually curated database that combines all publically available microarray gene expression data sets. Meta-analyses are performed by integrating gene expression data with clinical data. Patients are segregated into low vs. high expression cohorts, and Kaplan–Meier survival plots with log-rank *p* values are generated, along with hazard ratios and 95% confidence intervals. For the meta-analysis of *KCNH2* expression, we interrogated the KM plotter database for Affymetrix probe ID 210036_s_at, and the expression data were partitioned into tertiles. Analyses compared the top and bottom tertile groups

## Supplementary information

Supplementary Figure 1

Supplementary Figure 2

supplementary movie 1

supplementary movie 2

supplemental figure legends
