# Supplementary figures and images for: Potassium channel activity controls breast cancer metastasis by affecting β-catenin signaling
Source: Cell Death Dis. 2019 Feb 21;10(3):180. doi: 10.1038/s41419-019-1429-0 (PMC6385342; doi:10.1038/s41419-019-1429-0)

Supplementary Fig. 1

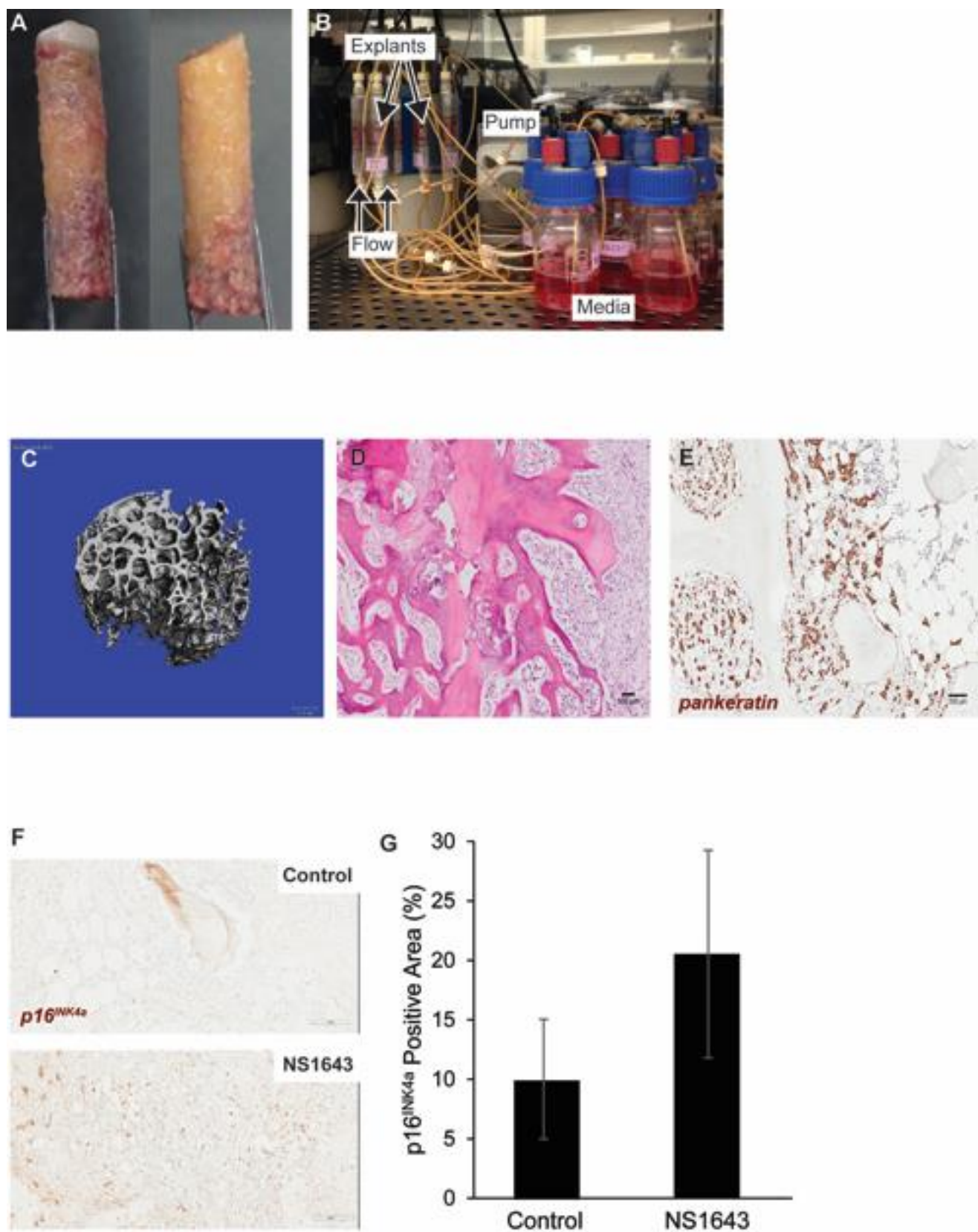

Supplement: Supplementary file 1 — Supplementary Figure 1 [file 41419_2019_1429_MOESM1_ESM.pdf]

Supplementary Fig. 2

B

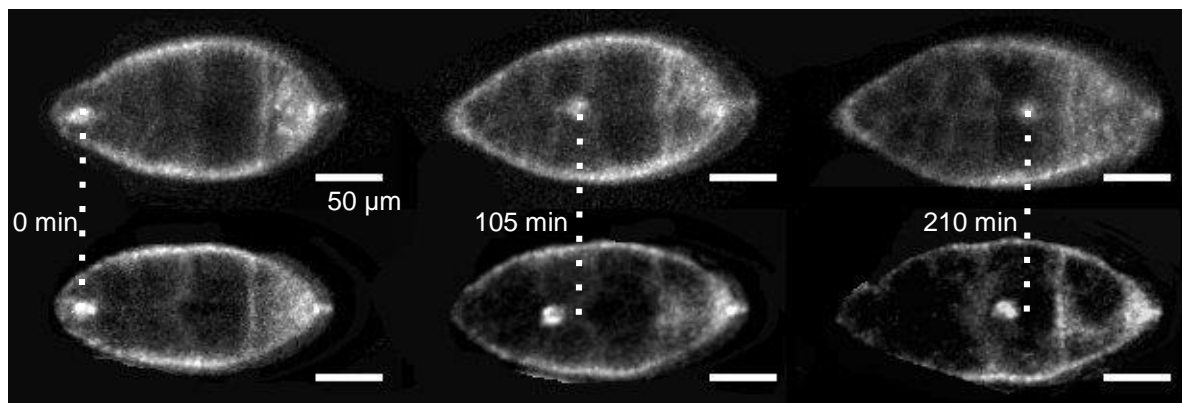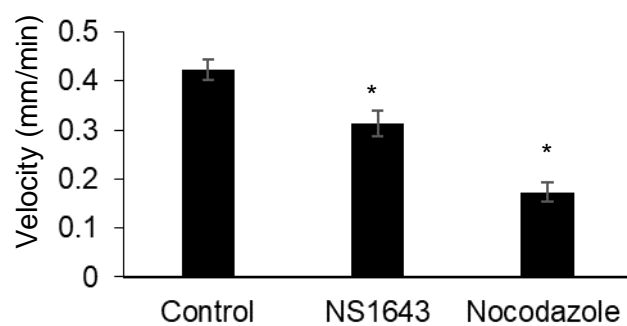

Supplement: Supplementary file 2 — Supplementary Figure 2 [file 41419_2019_1429_MOESM2_ESM.pdf]

## Slide 1
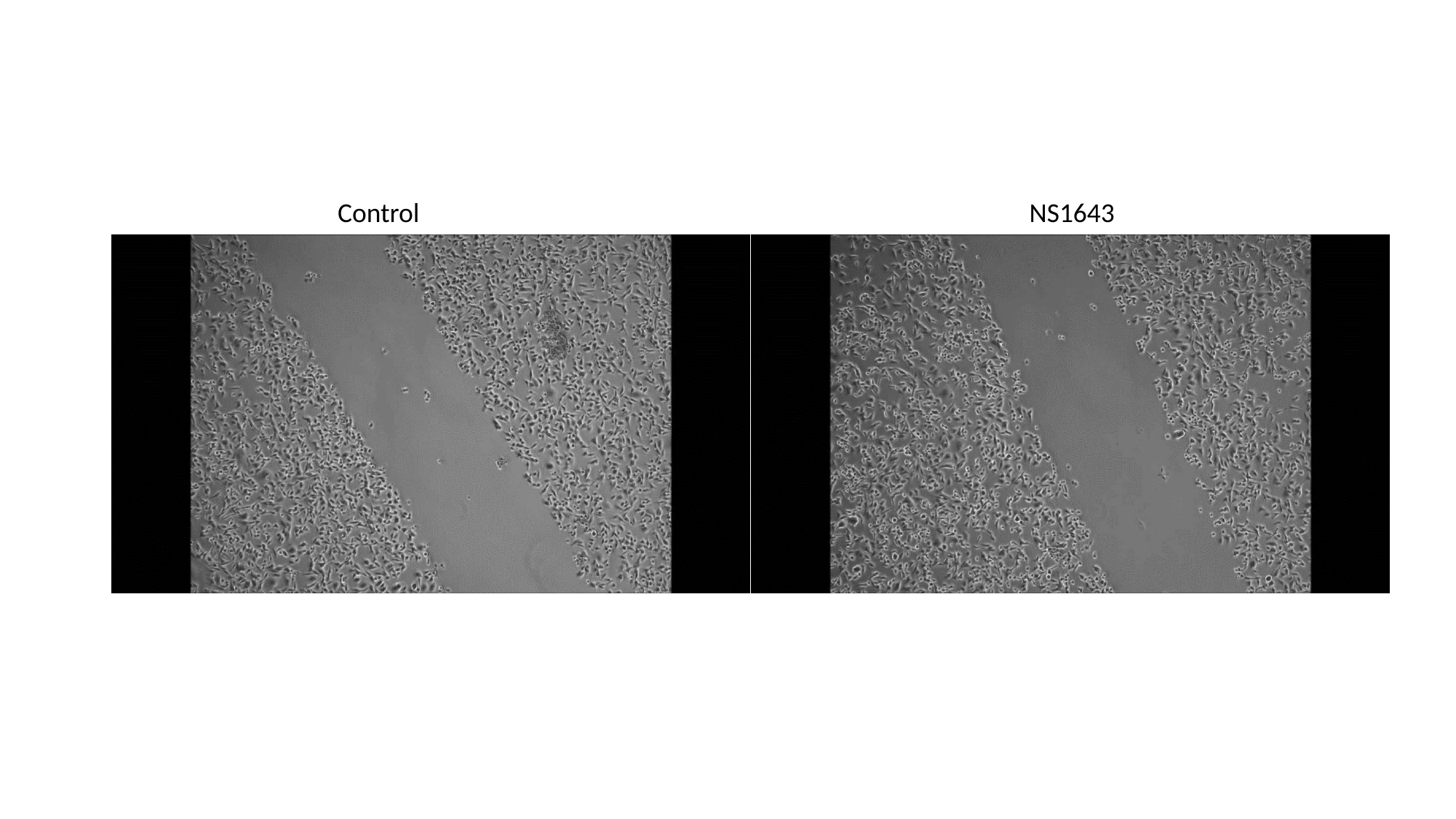

Control
NS1643

Supplement: Supplementary file 3 — supplementary movie 1 [file 41419_2019_1429_MOESM3_ESM.pptx]

## Slide 1
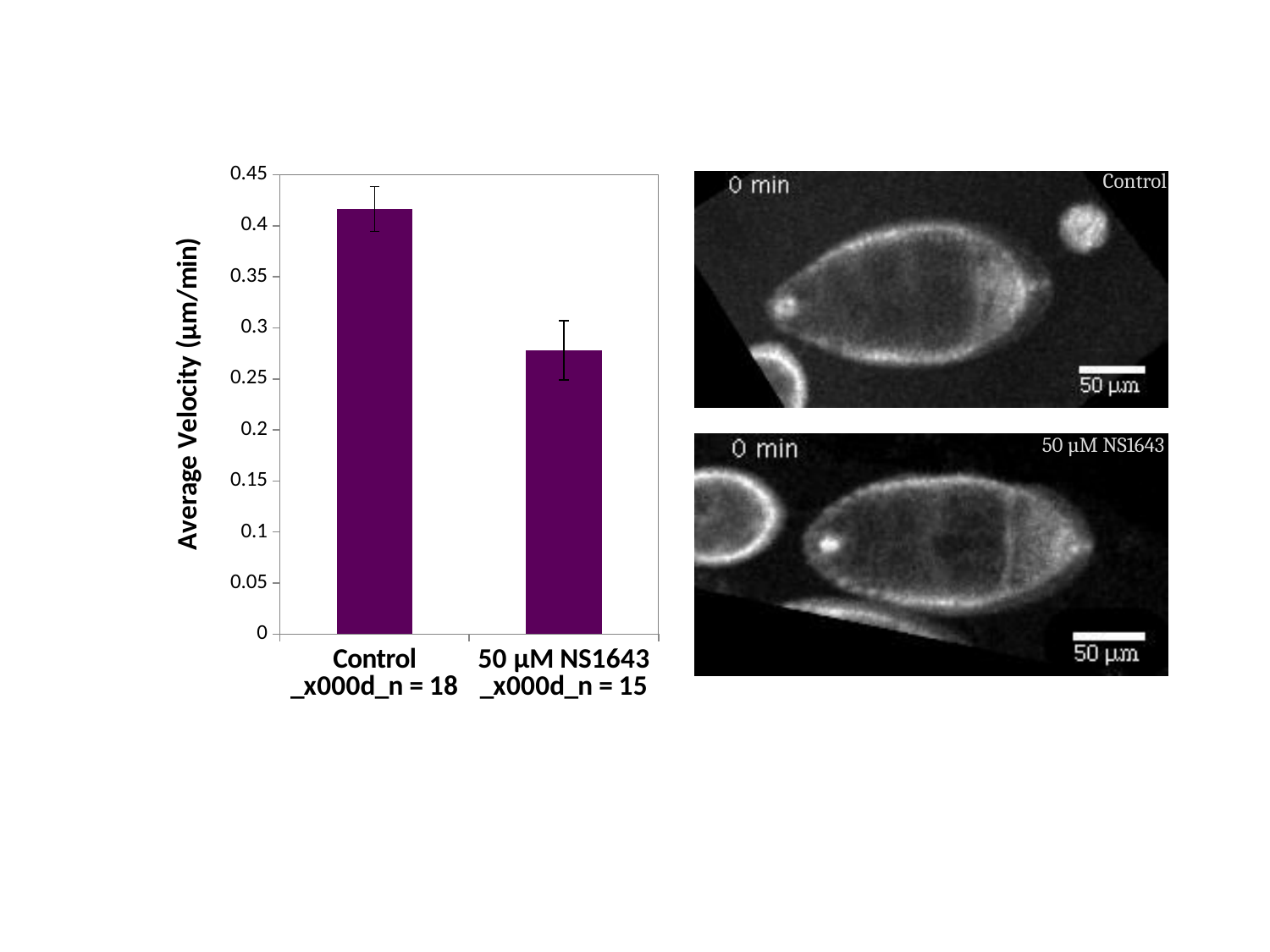

### Chart
| Category | Average |
|---|---|
| Control _x000d_n = 18 | 0.416533052566771 |
| 50 µM NS1643 _x000d_n = 15 | 0.278026350888775 |Control
50 µM NS1643

Supplement: Supplementary file 4 — supplementary movie 2 [file 41419_2019_1429_MOESM4_ESM.pptx]
