# Supplementary material for: Potassium channel activity controls breast cancer metastasis by affecting β-catenin signaling
Source: Cell Death Dis. 2019 Feb 21;10(3):180. doi: 10.1038/s41419-019-1429-0 (PMC6385342; doi:10.1038/s41419-019-1429-0)
Supplement: Supplementary file 5 — supplemental figure legends [file 41419_2019_1429_MOESM5_ESM.docx]

**Supplementary Figure 1. NS1643 suppresses the metastatic phenotype in breast cancer.**

A) Cylindrical explants were harvested using a diamond-tipped coring bit from a femoral head with lytic metastatic breast cancer lesions. B) Explants were maintained in a bioreactor culture system for 6 days either with or without 50 µM NS1643 supplementation. C and D) Micro-computed tomography (µCT) and H&E staining showed disorganized bone, lytic bone loss, and abnormal bone marrow morphology. E) Pan-cytokeratin staining indicated large regions of marrow containing cancer cells. F and G) Immunohistochemistry for p16^INK4a^ showed a trend toward the higher positively stained area in the NS1643 treated explants (p=0.1, n=4 per group; error bars show mean± S.D.).

**Supplementary Figure 2. NS1643 inhibits migration of somatic cells.** (A) Average net cluster velocity. Error bars represent standard error of the mean. Treatment with 50 µM NS1643 significantly inhibits the speed of border cell migration (two tailed t-test, p < 0.001). (B) Representative movies of carrier control (top) and 50 µM NS1643 (bottom).

**Supplementary Video information**

**Supplementary Video 1.** A) Movie of MDA-MB-231 cells treated with DMSO (control) or 50uM NS1643 (NS1643) immediately prior to scratch of monolayer. Images were taken every 5 minutes for 16 h. MDA-MB-231 cells were plated onto 6-well plates. Cells were incubated for 24 hours to allow a monolayer to form. Cells were scratched with a small pipette, 25 mM Hepes was added, and cells were treated immediately with 50uM NS1643 or DMSO. The sealed plate was then placed on the automated heated stage of an Olympus IX71 microscope set at 37°C and imaged with a 5X UPlanFLN objective lens. Images were collected using a Retiga SRV CCD camera, taking a frame every 5 min for 16 h from each of the wells using Image-Pro Plus software. Subsequently, all the acquired time-lapse sequences were displayed as a movie. Movies were tracked using ImageJ tracking plugin and analyzed using in-house Mathematica software as previously described (24). Only edge cells were tracked for entire 16-hour movie – data is two independent experiments 30 cells tracked per condition.

**Supplementary Video 2.** Movies of carrier control and 50μM NS1643.

Methods: Egg chambers from flies expressing E-cadherin::GFP were prepared and dissected as described previously (Prasad et al, 2007). Chambers were cultured using a slightly modified setup from Prasad, with 33 µg/mL human insulin, and using a Millicell® cell culture insert setup (described in Zartman et al 2013) in lieu of the lumox culture dish. Time-lapse movies were acquired by confocal microscopy with eleven 4 µm z-sections taken at 15 minute intervals. Analysis was done in FIJI using of egg chambers exhibiting normal morphology with a minimum duration of 1.5 hours. Net cluster migration was based on starting and final position of the cluster center. For drug treatment, culture media above the culture dish was supplemented with either 50 µM NS1643 or vehicle (DMSO). Imaging was set up immediately after dissection with an average lead time of about 20 minutes.
